# Compliance With Iron and Folic Acid Supplements Among Pregnant Women Attending Tertiary Care Hospital in the District of Peshawar

**DOI:** 10.7759/cureus.79495

**Published:** 2025-02-23

**Authors:** Mohammad Ummair, Aliya Durrani, Mahnoor Jamshaid, Marwah Amjad, Maria Gul, Jehan Z Khan, Abdul Qadeer Khan

**Affiliations:** 1 Department of Medicine, North Cumbria University Hospitals NHS Trust, Carlisle, GBR; 2 School of Medicine, Keele University, Staffordshire, GBR; 3 Department of Medicine, Peshawar Medical College, Peshawar, PAK; 4 Department of Gynecology, Ayub Teaching Hospital, Abbottabad, PAK; 5 Department of Pharmacy, CECOS University of IT and Emerging Sciences, Peshawar, PAK; 6 Department of Public Health, Khyber Medical University, Peshawar, PAK

**Keywords:** anemia, compliance, folic acid, iron, pregnant women

## Abstract

Objectives

This study aimed to determine the level of compliance with iron and folic acid supplementation (IFAS) and identify factors influencing compliance among pregnant women attending tertiary care hospitals in District Peshawar.

Methods

This cross-sectional study was conducted over a period of two months, from January 15, 2022, to April 30, 2022, among pregnant women visiting tertiary care hospitals in district Peshawar for antenatal checkups. A nonprobability-purposive sampling technique was used to collect the data. Pregnant women in their second and third trimesters were included, while those in the first trimester or with comorbidities were excluded. Data were collected using a validated and reliable questionnaire. Statistical analyses included the Chi-square test, independent t-test, and Fisher's exact test, where appropriate, with a significance level set at p < 0.05.

Results

The mean age of the study participants was 26.87 ± 6.80 years, with 24% residing in nuclear families. The majority of participants had attained at least a basic level of education. Obstetric history revealed an average of four pregnancies and two deliveries per participant, with 28.61% reporting previous abortions and 21.67% experiencing stillbirths. Antenatal care utilization was high, with 98% receiving prenatal care, while 52.78% had a documented history of anemia. Regarding IFAS compliance, only 34.4% of the participants met the adherence criteria. The primary motivators for compliance included encouragement from healthcare workers and fear of pregnancy-related complications. Conversely, forgetfulness and concerns about potential side effects were the most commonly cited barriers to adherence. Demographic factors associated with IFAS compliance included age, place of residence, family structure, participant’s education, spouse’s education, and median monthly income. In addition, pregnancy-related factors such as parity, total number of prenatal visits, gestational age at the initial visit, and the duration of supplement use during the current pregnancy were significantly associated with compliance. Multivariate logistic regression identified nuclear family structure as a significant predictor of lower compliance (OR = 0.513, 95% CI: 0.30-0.878, p = 0.015). Similarly, participants who took IFAS for only one month during pregnancy were significantly less likely to comply (OR = 0.246, 95% CI: 0.082-0.736, p = 0.012).

Conclusion

Low levels of compliance with iron and folic acid supplements were noted, highlighting a significant challenge. Family structure and the duration of IFAS intake during pregnancy play crucial roles in predicting compliance among pregnant women. Healthcare worker encouragement and addressing concerns about negative effects may enhance compliance. Interventions should be tailored to address these factors, which require immediate attention and resolution.

## Introduction

Anemia resulting from a deficiency in iron-folic acid is a pressing global public health concern, particularly prevalent in low- and middle-income countries. These essential micronutrients play a crucial role in physiological function, growth, and development for both the mother and newborn throughout pregnancy and after birth. This is also one of the causes of iron and folic acid deficiency contributing to 1.45% of all disability-adjusted life years (DALYs) [1,2]. The World Health Organization (WHO) recommends that a daily intake of 30-60 mg of iron and 0.4 mg of folic acid is essential for pregnant women to prevent adverse outcomes in the form of miscarriages, stillbirth, preterm birth, low birth weight, neural tube defects, preeclampsia, fetal malformation, puerperal sepsis, delayed psychomotor improvement, cognitive impairment, and a low score in newborns, which can have long-term detrimental effects. Overall, anemia is responsible for 18% of perinatal mortality, 19% of preterm births, and 12% of low birth weight in developing countries [3,4].

A recent report from the WHO reveals that approximately 32 million pregnant women worldwide are affected by anemia, with notable regional disparities in these figures. In high-income countries, prevalence rates are notably lower, with the United States reporting 18% and Australia 20%. In contrast, developing countries are experiencing a concerning upward trend, with Ethiopia at 50.1%, Sudan at 53%, Guinea at 71%, and Pakistan at a staggering 76.7% prevalence rate [5]. In alignment with the Universal Health Coverage (UHC) 2030 goal, aimed at accelerating equitable and sustainable global progress, maternal and child mortality remain two of the most critical health indicators. Addressing anemia and improving maternal health outcomes would significantly contribute to achieving UHC targets [6]. The most effective strategy for preventing and managing iron and folate-deficiency anemia during pregnancy is iron and folic acid supplementation (IFAS) with good compliance. However, studies from South Asia, Latin America, and parts of Africa indicate that compliance with IFAS remains low due to multiple factors. These include gastrointestinal side effects from iron intake, limited availability of supplements, inadequate counseling from healthcare professionals on proper usage and potential short-term side effects, and suboptimal utilization of prenatal healthcare services. Additionally, lack of awareness about the benefits of IFAS, along with cultural beliefs, attitudes, and perceptions, further contributes to poor adherence [7,8].

Although iron and folic acid supplements are provided free of charge in most countries through primary healthcare services, their utilization during pregnancy remains low. Several factors contributing to this issue remain unclear and require further investigation. This study aims to assess the level of compliance with IFAS and identify the associated factors among pregnant women in the Peshawar district.

## Materials and methods

Study design, duration, and study population

This cross-sectional study was conducted over a period of two months, from January 15, 2022, to April 30, 2022, among pregnant women attending both public and private tertiary care hospitals in Peshawar for antenatal checkups. Data were collected using a nonprobability purposive sampling technique. Pregnant women in their second and third trimesters were included, while those in the first trimester or with comorbidities were excluded.

Sample size

A total of 360 pregnant women were included in the study. The sample size was determined using the population proportion formula: n = Z² * P (1 - P) / E². The calculation was based on the following assumptions: an anticipated prevalence of 63.1%, derived from a previous study conducted in Karachi, Pakistan [9]; a margin of error of 0.05; and a standard normal deviation of 1.96 at a 95% confidence interval.

Ethical approval and consent

Ethical approval was obtained from the Prime Foundation Pakistan Ethical Review Committee (undergraduate) (reference no. Prime/ERC/2022-08; date: March 16, 2022). The study's purpose was explained to all participants, and informed written consent was obtained, ensuring data confidentiality.

Data collection tool

Data were collected using a structured, valid, and reliable questionnaire consisting of close-ended questions categorized into the following sections: socioeconomic and demographic information (age, marital status, place of residence, and educational status), obstetric factors (number of pregnancies and deliveries, history of stillbirths and abortions, antenatal visits, and history of anemia), and compliance with IFAS (level of compliance and noncompliance, along with the factors influencing them). Compliance with IFAS was defined as taking four or more tablets per week, while noncompliance was categorized as taking fewer than four tablets per week [10].

Statistical analysis

IBM SPSS Statistics for Windows, Version 25 (Released 2017; IBM Corp., Armonk, New York, United States) was used for data analysis. Descriptive statistics were applied to calculate the mean ± standard deviation (SD) or the median with interquartile range (IQR) for numerical variables and frequencies and percentages for categorical variables. Associations between categorical variables were assessed using the Chi-square test or Fisher’s exact test, as appropriate. Mean and median differences were analyzed using the Student’s independent t-test and the Mann-Whitney U test, respectively. Variables with a p-value of <0.2 in bivariate analysis were included in multivariate logistic regression to identify independent factors associated with compliance level. A significance level of 0.05 was considered.

## Results

Demographic characteristics of study participants

A total of 360 pregnant women participated in the study, with a mean age of 26.87 ± 6.80 years. Approximately 24% lived in nuclear families, while 273 (75.83%) were part of extended families. The majority of respondents were educated (208, 57.8%), while 152 (42.22%) had no formal education. Additionally, 64 (17.78%) participants had illiterate partners, whereas 296 (82.3%) had educated partners. The average recorded monthly income was 30,000 Pakistani Rupees (PKR).

Obstetric and health-related characteristics of the study population

Each respondent reported an average of four pregnancies (median: 4 (IQR: 3)) and two deliveries (median: 2 (IQR: 2)). A total of 103 (28.61%) had experienced a previous abortion, while 78 (21.67%) had endured a stillbirth. Approximately 98% of participants had received antenatal care for their most recent pregnancy, with the majority (261, 72.50%) initiating care during the first trimester. Anemia had affected 52.78% of the women in the past, while 209 (58.06%) had experienced it during their current pregnancy.

Compliance to IFAS

**Figure 1:**
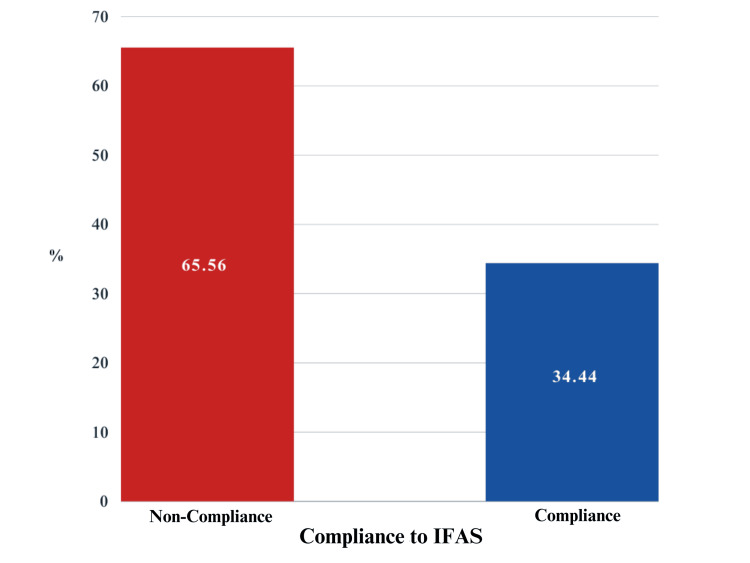
Compliance level to IFAS among pregnant women IFAS: iron and folic acid supplementation

Regarding supplement compliance, 124 participants (34.4%) met the criteria for iron and folic acid intake, as shown in Figure 1.

**Figure 2:**
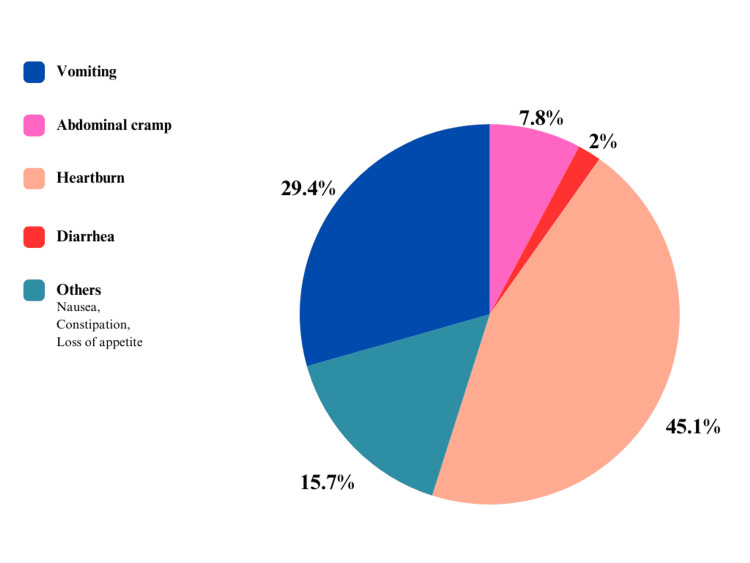
Side effects of IFAS reported by the study participants IFAS: iron and folic acid supplementation

As shown in Figure 2, the study participants reported two main factors influencing compliance. First, 47 participants (37.9%) stated that healthcare workers encouraged them to take the supplements correctly. Second, 37 participants (29.8%) cited a fear of becoming ill as a motivating factor.

**Figure 3:**
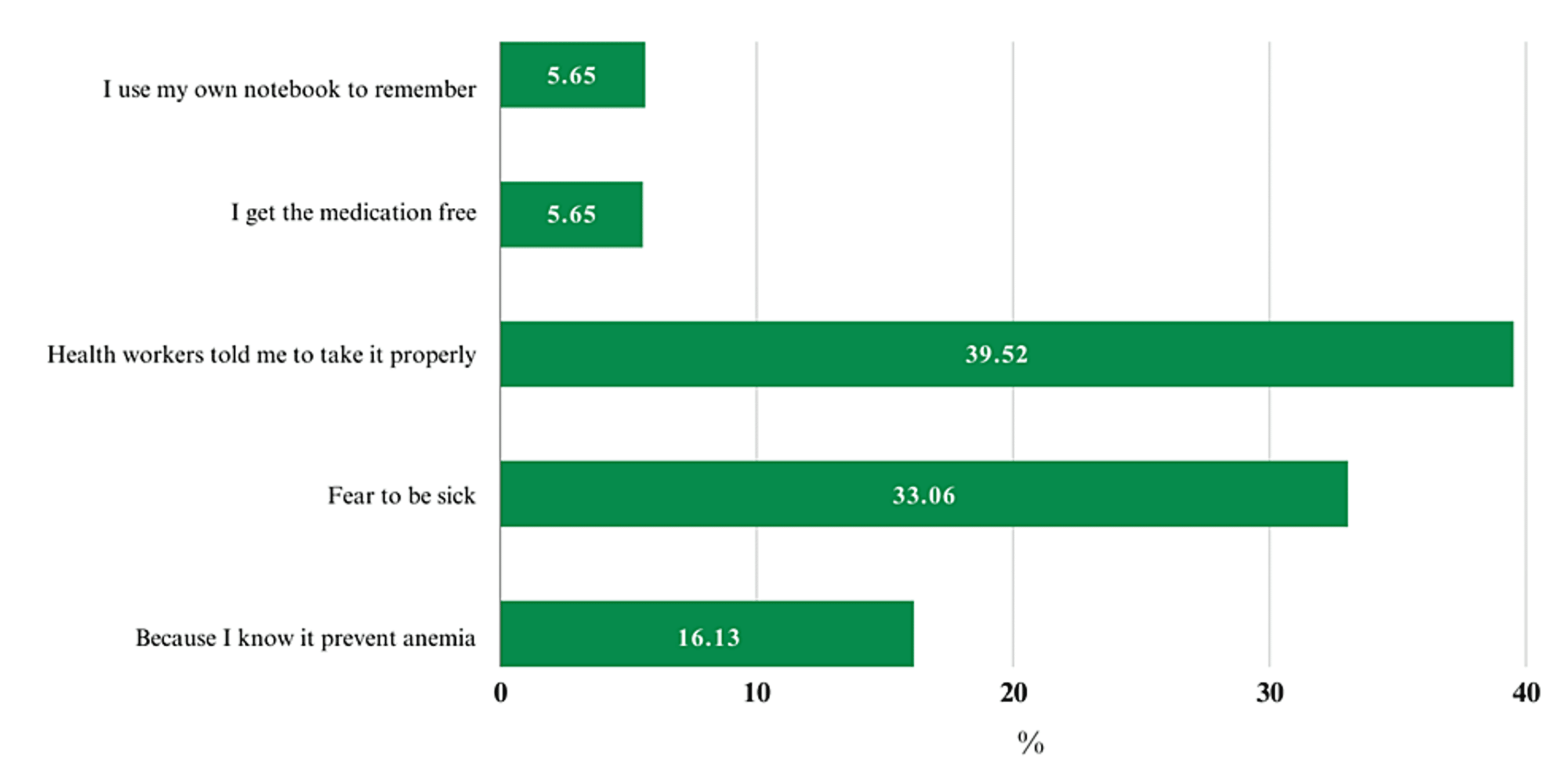
Factors associated with compliance to IFAS among pregnant women IFAS: iron and folic acid supplementation

Similarly, factors analyzed as responsible for noncompliance showed that nearly 65% of people did not follow the prescribed course of action, with 85 (36.01%) of them forgetting to take the supplements. Another significant reason was that 47 (19.91%) of them avoided the supplements out of concern for potential negative effects (Figure 3).

**Figure 4:**
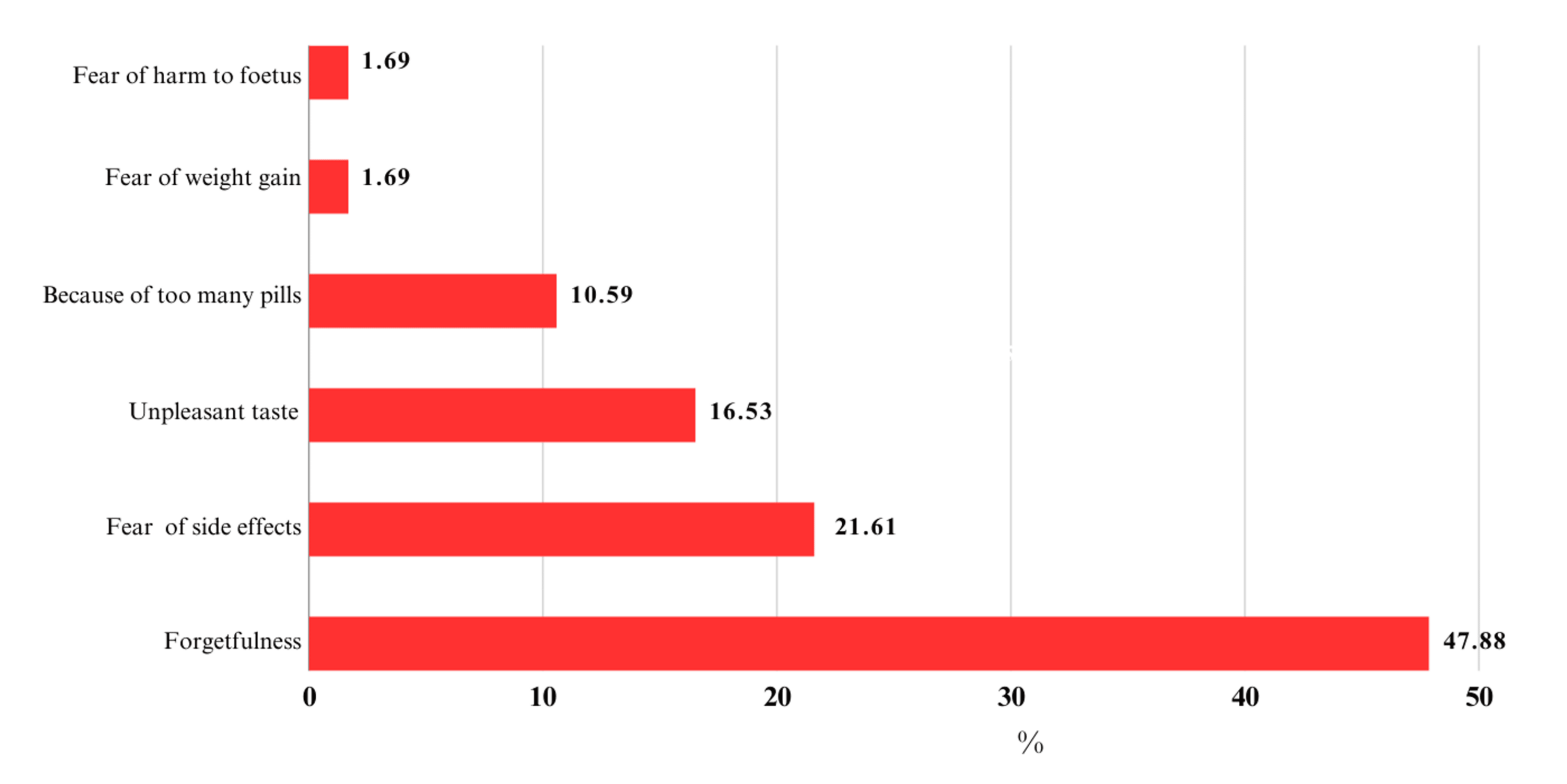
Factors associated noncompliance with IFAS among pregnant women IFAS: iron and folic acid supplementation

Factors associated with noncompliance to IFAS among the study sample are shown in Figure 4. 

The Chi-square test, Student's independent t-test, Mann-Whitney statistics, and Fisher's exact test were used to determine the association between compliance with IFAS and demographic and obstetric factors. The findings showed that demographic factors such as age (p = 0.042), residence (p = 0.002), family structure (p = 0.005), participant's and spouse's education (p < 0.01), and average monthly income (p = 0.030) were related to IFAS compliance as depicted in Table 1.

**Table 1:** Association of demographics characteristics with compliance with IFAS among pregnant women IFAS: iron and folic acid supplementation; IQR: interquartile range

Characteristics		Compliance		p-value
		<4	>4	
Age, mean ± SD		27.39 ± 7.19	25.86 ± 5.90	0.042
Residence, n (%)	Rural	77 (32.63)	59 (47.58)	0.005
	Urban	159 (67.37)	65 (52.42)	
Marital status, n (%)	Married	232 (98.31)	123 (99.19)	0.494
	Divorced	4 (1.69)	1 (0.81)	
Family system, n (%)	Nuclear	45 (19.07)	42 (33.87)	0.002
	Extended	191 (80.93)	82 (66.13)	
Participant education, n (%)	No formal education	110 (46.61)	42 (33.87)	0.018
	Primary	36 (15.25)	20 (16.13)	
	Secondary	58 (24.58)	30 (24.19)	
	Above secondary	32 (13.56)	32 (25.81)	
Participants’ husband education, n (%)	No formal education	50 (21.19)	14 (11.29)	0.007
	Primary	42 (17.8)	13 (10.48)	
	Secondary	66 (27.97)	38 (30.65)	
	Above secondary	78 (33.05)	59 (47.58)	
Family monthly income, median (IQR)		30000 (20000)	25000 (25000)	0.030

Similarly, obstetric and pregnancy-related characteristics indicated that parity (p = 0.005), the total number of prenatal visits (p = 0.008), gestational age at the initial visit (p < 0.001), and the length of supplement use during the current pregnancy (p < 0.001) were among the factors associated with compliance with IFAS as given in Table 2.

**Table 2:** Association of obstetric characteristics with compliance with IFAS among pregnant women IFAS:  iron and folic acid supplementation

Characteristics		Compliance		p-value
		<4	>4	
Gravida	Primagravida	32 (13.56)	25 (20.16)	0.103
	Multigravida	204 (86.44)	99 (79.84)	
Parity	Nullpara	11 (4.66)	12 (9.68)	0.005
	Primapara	44 (18.64)	37 (29.84)	
	Multipara	181 (76.69)	75 (60.48)	
History of stillbirth	Yes	55 (23.31)	23 (18.55)	0.298
	No	181 (76.69)	101 (81.45)	
Number of stillbirths, median (IQR)		1.00 (0.00)	1.00 (0.00)	0.581
History of abortion	Yes	68 (28.81)	35 (28.2)	1.000
	No	168 (71.19)	89 (71.77)	
The total number of abortions, median (IQR)		1.00 (1.00)	1.00 (1.00)	0.837
Received antenatal care for recent pregnancy?	Yes	230 (97.46)	124 (100)	0.097
	No	6 (2.54)	0 (0)	
Total number of antenatal care visit, median (IQR)		4.00 (1.00)	4.00 (0.00)	0.008
Gestational age at first antenatal care	First trimester	155(65.68)	106(85.48)	<0.001
	Second trimester	36 (15.25)	13 (10.48)	
	Third trimester	45 (19.07)	5 (4.03)	
History of anemia in a previous pregnancy	Yes	126 (53.39)	64 (51.61)	0.748
	No	110 (46.61)	60 (48.39)	
History of anemia in current pregnancy	Yes	142 (60.17)	67 (54.03)	0.262
	No	94 (39.83)	57 (45.97)	
Duration of intake of supplements during the current pregnancy?	One month	42 (17.8)	4 (3.23)	<0.001
	Two months	32 (13.56)	4 (3.23)	
	Three months	31 (13.14)	12 (9.68)	
	>Three months	131 (55.51)	104 (83.87)	
If the answer to <4 supplements was a side effect, what was that?	Vomiting	15 (30.61)	0 (0)	0.639
	Diarrhea	1 (2.04)	0 (0)	
	Heartburn	21 (42.86)	2 (100)	
	Abdominal cramp	4(8.16)	0 (0)	
	Other	8 (16.33)	0 (0)	

Parameters demonstrating biological plausibility or a statistical significance of p < 0.2 in bivariate analyses were included in the multivariate logistic regression to identify independent factors associated with compliance. The analysis identified a nuclear family structure as a significant predictor of lower compliance (OR = 0.513, 95% CI: 0.30-0.878, p = 0.015). Similarly, participants who took IFAS for only one month during pregnancy were significantly less likely to comply (OR = 0.246, 95% CI: 0.082-0.736, p = 0.012).

## Discussion

Our investigation found that 34.4% of study participants exhibited IFAS compliance. This result is significantly lower than that reported in a study conducted in Ethiopia, a low-income nation, where the compliance rate was 40.9%, and partner support and awareness of IFAS were identified as key drivers of compliance [11]. In 2021, a survey conducted in a different region of Ethiopia revealed a substantially higher level of compliance than the earlier study, at 71.8%. In this case, healthcare providers' recommendations for the IFAS were identified as influential in increasing compliance. Despite both studies being hospital-based, a significant variation in compliance level was observed, possibly attributed to the development of IFAS awareness initiatives through various social media channels [10].

In India, a similar study was conducted, revealing a significantly higher compliance rate of 64.7% compared to our study. Here, the main obstacles to noncompliance were forgetfulness, and the reported and observed adverse effects were associated with IFAS therapy. The reasons for the noncompliance in this instance align with our study. Similar survey results in Kenya show a compliance rate of 32.7%, indicating that it is a serious issue in the area [6].

A cross-sectional survey on the causes of antenatal IFAS nonuse among women was conducted in 14 districts of Pakistan; the results revealed a compliance rate of 38.3%, slightly higher than observed in our study. In this study, nearly two-fifths of the women who initiated IFAS during their second trimester obtained the supplements from a community health professional. The three primary causes of noncompliance were identified as illiteracy, being older than 45, and lack of access to antenatal care services [12]. Comparative studies from other regions present varying compliance rates and factors influencing IFAS usage. In Karachi, a study reported a notably higher compliance rate of 63.1% despite similar reasons for noncompliance, such as forgetfulness and fear of side effects [9]. Conversely, in Tanzania, the compliance rate among expectant women with IFAS was lower at 20.3%, with factors like delayed antenatal care visits, distance to healthcare facilities, the presence of IFAS at the health center, and inadequate counseling contributing to noncompliance. Notably, forgetfulness and fear of side effects were shared factors in our study [13]. In India, a significantly high compliance rate of 85.7% was observed, with parameters linked to compliance including participant education, income, number of antenatal visits, and supplement duration, mirroring our study findings [14]. Similarly, in Iran, compliance rates for iron and folate were relatively high, at 71.6% and 81.5%, respectively. Forgetfulness and fear of side effects were consistent factors for noncompliance, consistent with our study findings. Age and educational attainment were also identified as statistically correlated factors in both iron and folate compliance, similar to our observation [15]. A contrasting scenario was noted in northwest Ethiopia, where the IFAS compliance rate in 2017 was relatively low at 28.7%; however, after two years, it increased to 47.6%, with forgetfulness and fear of side effects remaining consistent reasons for noncompliance [16,17]. This suggests the dynamic nature of IFAS compliance and the potential impact of interventions over time.

Recommendations

Every health center should maintain an adequate supply of IFAS supplements. All expectant women should be encouraged to undergo routine antenatal care examinations, which should include hemoglobin estimation and completion of the recommended treatment. It is crucial to inform expectant mothers about the hazards of untreated anemia and the typical adverse effects of IFAS and dispel widespread misconceptions about taking IFAS tablets. IFA supplements must be taken according to the provided directions, and incorporating iron-rich foods into their diet is essential.

## Conclusions

Overall, low levels of compliance with iron and folic acid supplements were noted, highlighting a significant challenge. Family structure and the duration of IFAS intake during pregnancy play crucial roles in predicting compliance among pregnant women. Healthcare worker encouragement and addressing concerns about negative effects may enhance compliance. Interventions should be tailored to address these factors, which require immediate attention and resolution.
